# Adherence to glucose monitoring with intermittently scanned continuous glucose monitoring in patients with type 1 diabetes

**DOI:** 10.1007/s12020-022-03288-1

**Published:** 2022-12-27

**Authors:** Carolina Sousa, João Sérgio Neves, Cláudia Camila Dias, Rute Sampaio

**Affiliations:** 1 Departament of Biomedicine, Faculty of Medicine of the University of Porto (FMUP), Porto, Portugal; 2 Department of Physiology and Cardiothoracic Surgery, Faculty of Medicine of the University of Porto (FMUP), Porto, Portugal; 3 Department of Endocrinology, Metabolism and Diabetes, Centro Hospitalar Universitário de São João, Porto, Portugal; 4 CINTESIS@RISE, MEDCIDS, Faculty of Medicine of the University of Porto, Porto, Portugal; 5 Knowledge Management Unit and Department of Community Medicine, Information and Health Decision Sciences (MEDCIDS), Faculty of Medicine of the University of Porto (FMUP), Porto, Portugal

**Keywords:** Adherence, Type 1 Diabetes, Intermittently scanned continuous glucose monitoring (isCGM), Illness representations, Treatment representations

## Abstract

**Purpose:**

This study aims to predict the Intermittently scanned continuous glucose monitoring (isCGM) adherence behavior of patients with Type 1 Diabetes.

**Methods:**

Patients with Type 1 Diabetes mellitus using FreeStyle Libre™ System (FL), a isCGM device, that attended the “Insulin Infusion Pump clinic” at *Centro Hospitalar de São João* were enrolled and evaluated for sociodemographic and clinical characterization, beliefs and concerns about Diabetes Mellitus, as well as isCGM’s perceptions. Intermittently scanned continuous glucose monitoring data were collected to characterize monitoring patterns and to measure isCGM’s adherence—FL average of scans/day.

**Results:**

Seventy-two patients with a mean of 30.36 years (sd=11.35) participate in this study. A median of 7 scans/day was performed. The adherence predictors found was Age (*β* = 0.191, *p* = 0.006), Time in target (*β* = 0.530, *p* = 0.002), isCGM Necessity (*β* = 2.631, *p* = 0.048), Body Mass Index (*β* = −0.549, *p* = 0.017) and Sex (*β* = −3.996; *p* = 0.011).

**Conclusions:**

This study emphasizes the relevance of glucose monitoring adherence in disease control and shows that males of younger ages, presenting with higher body mass index levels, lower time in target, and reporting lower isCGM necessity are less adherent to isCGM. Therefore, these patients should be closely followed and object of personalized strategies to promote treatment adherence.

## Introduction

Diabetes is a worldwide health concern, affecting more than 536 million patients in 2021, and is estimated to reach 783 million in 2045 [1]. In Portugal, this condition is particularly prevalent, as in the latest data available in 2018, it affects 13.6% of the Portuguese population between 20 and 79 years, representing 8% of Portugal´s health expenses [2].

Treatment adherence is essential to maintain adequate metabolic control and avoid diabetes complications. Therapeutic adherence is defined as “the extent to which a person’s behavior (in terms of taking medications, following diets, or executing lifestyle changes) coincides with medical or health advice” [3]. In chronic conditions such as diabetes, treatment usually follows a complex plan which combines the patient’s education to recognize hyper and hypoglycemia symptoms [4] and self-care behaviors—such as glucose monitoring, diet, medication, regular physical activity, foot care, and regular medical visits [5]. These therapeutic interventions require the commitment of the patient and an active role in managing his or her disease [6].

Measuring treatment adherence in diabetes can be challenging, as it must assess the adherence level in each treatment regimen independently component [3]. This brings challenges like the scarcity of appropriate methods to evaluate adherence to insulin treatment [7] or variations in recommended blood glucose monitoring frequencies for patients with type 1 diabetes [3]. Hence, some studies showed low adherence to different therapy components [3, 8, 9].

Frequent glucose monitoring is recommended for patients treated with insulin – all patients with type 1 diabetes mellitus (T1D) and some patients with type 2 diabetes mellitus (T2D) - so they can adjust insulin dose, depending on factors such as diet and exercise [10]. For many years, patients had to use test strips and finger-stick blood samples to monitor glucose levels [10]. However, this method has barriers that can be reduced with continuous glucose monitoring (CGM) and intermittently scanned continuous glucose monitoring (isCGM), particularly benefiting patients unable or unwilling to self-monitor blood glucose (SMBG) due to pain or discomfort [11]. FreeStyle Libre™ System (FL) is an isCGM that also overcomes some CGM barriers. It doesn´t constantly update the glucose measurements, like CGM, but the current glucose value is quickly available when needed [12]; it doesn’t require calibration; has an extended sensor lifetime of 14 days; and is relatively affordable [13]. These advantages may explain patient satisfaction and the market expansion of this device [13]. It is important to emphasize that isCGM also shows higher monitoring rates when compared to SMBG [13, 14], which can be associated with improved glycaemic control [13].

Therefore, given its increasing usage, it´s urgent to understand the adherence patterns of isCGM´s users and establish possible associations with sociodemographic and clinical variables, isCGM monitoring variables, believes and concerns about Diabetes Mellitus, and isCGM perceptions. More specifically, using a conceptual framework of Illness and Treatment Representations [15] (which focuses on how a patient’s beliefs and expectations about an illness determine a person’s appraisal and coping with perceiving it as manageable or threatening) and understanding the Necessities versus Concerns [16, 17] about self-monitoring, this study aims at identify patients that won´t adhere to isCGM and why, so clinicians can implement strategies to enhance its use and reduce diabetes morbidity.

## Methods

### Study design

This is an observational and retrospective study performed between 20th October 2021 and 1st June 2022 at the *Centro Hospitalar de São João, E.P.E. - Hospital de São João*.

### Participants

The participants were adult patients with Type 1 Diabetes mellitus using FreeStyle Libre (FL), who presential attended the “Insulin Infusion Pump clinic” and signed the informed consent to participate. The exclusion criteria were: inability to communicate in Portuguese; presenting psychiatric and cognitive disorders precluding the interview; using other devices for reading FL; less than a month since FL’s first prescription; and without records on LibreView System.

Of the 158 patients followed in the “Insulin Infusion Pump clinic”, 91 met the inclusion criteria. Of these, 1 refused and 18 patients were excluded: 13 for using other devices to read the FL, 4 with missing information in LibreView; 1 with less than one month since the FL prescription.

### Data collection

Data was collected by asking direct clinical questions to patients and from medical records, by application of questionnaires and Libre View consultation, respectively. All participants were informed about the study objectives and data collection procedures before being invited to sign an informed consent form which included authorization to use the gathered information.

### Instruments

A questionnaire was given to all the participants. It addressed sociodemographic and clinical questions, questions based on the Brief Illness Perception Questionnaire (Brief IPQ) [18], and an Intermittently scanned Continuous Glucose Monitoring questionnaire based on some of the factors that contribute to adherence [3]. Were also collected isCGM Monitoring Data by LibreView platform.

#### Sociodemographic questionnaire

Information about sex, age, marital status, education level, and the professional situation was gathered using this questionnaire.

#### Clinical questionnaire

Data about diabetes course- age at diagnosis and T1D duration, comorbidities, hospital or emergency department admissions; body mass index (BMI); method of insulin administration (pump/pen); HbA1c at the consult, was obtained using a structured clinical questionnaire. To complete the missing data, medical records were used.

#### Brief illness perception questionnaire (Brief IPQ) [18]

Brief IPQ is a validated nine-item scale designed to rapidly assess illness’s cognitive and emotional representations [18], using a 10-point Likert scale, with a total of eight items. Five of the items assess cognitive illness representations: consequences (item 1), timeline (2), personal control (3), treatment control (4), and identity (5); two of the items assess emotional representations: concern (6) and emotions (8); one item assesses illness comprehensibility (7). High scores (total result) reveal a more threatening perception of the illness.

In the present study, we did not use the timeline (item 2), as diabetes is a chronic disease without a fully understood etiology related to genetic and environmental factors [3]. Treatment control (Item 4) also wasn´t implemented as insulin is crucial and the only available treatment.

#### Intermittently scanned continuous glucose monitoring questionnaire

Based on the dimensions defined by WHO to understand the phenomenon of adherence [3], information about some of the most notable therapy-related factors was collected by a 5-point Likert scale questionnaire, namely: Information about isCGM; isCGM efficacy and sufficient in disease control; isCGM necessity; Patient satisfaction with isCGM monitoring; self-efficacy in isCGM monitoring; concerns with isCGM use; familiar support in isCGM monitoring. It was also asked about the individual perception of the average frequency of isCGM scans per day and if they consider that number enough.

#### Intermittently scanned continuous glucose monitoring data

In this study, it was used the FreeStyle Libre device for glucose monitoring, and to access monitoring data, the LibreView platform. This platform keeps the information about glucose monitoring and patterns in a cloud so professionals and patients can access their account and create reports, facilitating diabetes follow-up.

On the day of the medical appointment, data from the previous 28 days were accessed: the average scans/day with FreeStyle Libre; percentage of time in glucose target, below and above; average glucose; the number of low glucose events; and glucose variability. As the FL sensor has a 14 days lifetime, to prevent possible bias with sensor application adherence, patients with a gap between sensors exchange higher than three days, we collected data only from the 14 days after the sensor application instead of 28 days.

The average of scans/day with Freestyle Libre- monitoring frequency- was used as an accurate way to measure adherence to isCGM.

### Ethics

This study had the permission of the *Centro Hospitalar de São João* Ethics Committee, with approval number 254/21, and all patients enrolled gave their written consent after they were given the information about the study.

### Statistical analyses

Categorical variables were described as absolute frequencies (n) and relative frequencies (%). Medians and percentiles were used for continuous variables.

When testing a hypothesis about continuous variables, nonparametric tests Mann–were used as appropriate, taking into account normality assumptions and the number of groups compared; when testing a hypothesis about categorical variables, a chi-square test and Fisher’s exact test were used, as appropriate. To understand the adherence patterns of isCGM´s users and identify possible associations with sociodemographic and clinical variables, univariate and multivariate linear regression modeling was used. Coefficient regression (beta), 95% confidence intervals (95% CI) and R^2^ as a measure of goodness of fit were presented. Models were built according to the backward stepwise approach.

The significance level used was 0.05.

Statistical analysis was performed using the software Statistical Package for the Social Sciences v. 27.0.

## Results

### Sociodemographic characterization

The socio-demographic data is presented in Table 1. The final sample has 72 patients, 29 males and 43 females. The age of the participants ranged from 18 to 66, with a mean of 30.36 years (sd=11.35). The majority of patients completed high school or college (93.1%), had a full-time job (65.3%), and were single (57.7%).

**Table 1:** Sociodemographic and clinical data

Variable	
Sex, n(%)	
Male	29 (40.3)
Female	43 (59.7)
Age	
Range (years)	18–66
Mean (SD)	30.4 (11.4)
Median (P25-P75)	25.50 (21–37)
Years n(%)	
18–29	39 (54.2)
30–39	18 (25)
40–49	9 (12.5)
≥50	6 (8.3)
Education, n(%)^a^	
Low	5 (6.9)
High	67 (93.1)
Profession, n(%)	
Student	21 (29.2)
Unemployed	3 (4.2)
Full Time Job	47 (65.3)
Retired	1 (1.4)
Marital Status, n(%)^b^	
Single	41 (57.0)
Married	30 (41.6)
Insulin administration, n(%)	
Pen	17 (23.6)
Pump	55 (76.4)
BMI	
Range (kg/m^2^)	18.3–32.0
Mean (SD)	24.6 (3.4)
Median (P25-P75)	23.71 (22.0–27.0)
Categories n(%)^c^	
Underweight/ Normal	42 (58.3)
Overweight	22 (30.6)
Obese	8 (11.1)
Age at diagnosis	
Mean (SD)	13.7 (9.4)
Median (P25-P75)	11 (8–19)
T1D duration (years)	
Mean (SD)	16.6 (8.3)
Median (P25-P75)	15.5 (9.2–22.0)
HbA1c	
Mean (SD)	7.51 (0.96)
Median (P25-P75)	7.3 (6.9–8.1)
Past year hospital or ED admissions, n (%)	
Yes	7 (9.7)
No	65 (90.3)
Diabetes comorbidities, n (%)	
Yes	15 (20.8)
No	57 (79.2)

*BMI* body mass index, *T1D* type 1 diabetes, *ED* emergency department, *SD* standard deviation, *P25-P75* 25th percentile and 75th percentile (representing the interquartile range)

^a^Low: up to 9 grade

^b^One non response

^c^Underweight/ Normal <25 kg/m^2^; Overweight 25–29,9 kg/m^2^; Obese >29,9 kg/m^2^

### Clinical characterization

The clinical characterization is presented in Table 1. All participants were followed at the “Insulin Infusion Pump clinic”, however, only 55 (76.4%) used an insulin pump, and the remaining 17 patients (23.6%) used insulin pens. The majority had a low or normal BMI (58.3%), 30.6% of the patients had overweight, and 11.1% had obesity. The mean age at diagnosis was 13.7 years and T1D duration was 16.6 years. HbA1c had an average of 7.5%. When asked if they had diabetes-related comorbidities, 20.8% responded yes, and 9.7% of the patients reported hospital or emergency department (ED) admissions in the previous year.

### Intermittently scanned continuous glucose monitoring data (isCGM)

The median (P25-P75) percentage of time in glucose target, above and below target, was respectively 51% (39.25–66), 43% (28–56) and 4% (2–7). The patient’s average glucose had a median of 172 mg/dL (149–199), with a variation of glucose of 40.65% (35.7–44.4) and a median number of low glucose events of 15 (6–26).

### Adherence patterns of isCGM

IsCGM adherence was measured by the FL monitoring frequency of the patient, and it showed a median (P25-P75) of 7 scans/day (5–12).

There were not significant differences between monitoring frequency and sociodemographic characteristics of the sample. Neither the presence of comorbidities, the ED or hospital admissions, nor the way of insulin administration had significant monitoring frequency differences between groups. However, patients with obesity had a lower monitoring frequency than underweight/normal BMI (*p* = 0.012), with a median of 3.5 versus eight scans/day.

The perceived adherence to isCGM monitoring, given by the average perceived number of scans per day, had a median (P25-P75) of 8 scans/day (5.25–10), where 11.3% of the patients had the same number of perceived and effective scans, but 40.8% underestimate and 47.9% overestimate this number. Patients that overestimated the adequate number of scans/day had higher BMI levels (*p* = 0.005), higher HbA1C (*p* = 0.028), and lower scores on the emotional item (*p* = 0.011) than the ones that underestimated this number. The majority of the patients, 68.1%, believed the number of scans/day performed was enough for diabetes control. This same group had higher isCGM adherence when compared to patients who believed their number of scans was not enough (*p* = 0.016).

When analyzing the association between monitoring frequency and different study variables, it was found a positive and statistically significant association with age (*β* = 0.21, *p* = 0.011) and percentage of time in glucose target (*β* = 0.28, *p* < 0.001); and also a negative and significative relation with HbA1c (*β* = −3.43, *p* < 0.001), BMI (*β* = −0.62, *p* = 0.025), average glucose (*β* = −0.09, *p* < 0.001), percentage of time with glucose above target (*β* = −0.22, *p* < 0.001) and glucose variability (*β* = −0.34, *p* = 0.013).

### Cognitive and emotional representations of diabetes and adherence to isCGM

The assessment of cognitive diabetes representation resulted in a median (P25-P75) score of 5 (3–6) points for the disease consequences representation, 3 (2–4) in personal control and 5.50 (4–7) in identity; illness comprehensibility had a median score of 2 (1–2); the emotional representation of diabetes resulted in a median score of 8 (6–9) in disease concern and 6 (2–8) in emotional item.

It was not found a significant difference between cognitive or emotional illness representation and sociodemographic variables. However, patients with underweight/normal BMI showed better diabetes comprehensibility, with a median score of 9 points, when compared to patients with overweight or obesity, that had both a median of 8 points, respectively *p* = 0.013 and *p* = 0.011. The presence of comorbidities or how insulin is administered didn’t show differences in illness representation. However, patients with ED or hospital admissions in the past year had higher scores on cognitive illness representation, revealing them to be more threatened by diabetes (*p* = 0.007).

When adjusting for Brief IPQ questions, a statistically significant and positive association was found between monitoring frequency and the perception of illness consequences (*β* = 1.07, *p* = 0.017).

### Perceived isCGM necessity and concerns and adherence to isCGM

Patients gave a median (P25-P75) score of 5 points for isCGM necessity (4–5); 4 for isCGM monitoring information (4–5), efficacy and sufficiency (4–4), self-efficacy (4–5), satisfaction (4–5), and familial support (3–5); and 2 for concern (1–3).

When analyzing differences between perceived isCGM necessity and concerns and sociodemographic and clinical characteristics, it wasn´t found relevant associations.

When adjusting for isCGM monitoring questions, a statistically significant association was found between monitoring frequency- adherence- and the isCGM necessity (*β* = 4.825, *p* = 0.002) and satisfaction (*β* = −2.57, *p* = 0.037).

### Predictors of adherence to isCGM

A multivariate regression was performed, adjusting for variables: Sex, BMI, Age, HbA1c, Time in target, Time above target, Average glucose, Glucose variability, Illness consequences and identity, isCGM necessity, and isCGM satisfaction. The results of this analysis are presented in Table 2.

**Table 2:** Predictors of adherence to isCGM

Measurements	β	CI 95% [Inferior, Upper Limit]	*p*
Sex			
Female	Ref		
Male	−3.996	[−7.029, −0.962]	0.011
BMI, kg/m^2^	−0.549	[−0.995, −0.103]	0.017
Age, years	0.191	[0.057, 0.325]	0.006
Time in target, %	0.530	[0.196, 0.864]	0.002
Time above target, %	0.290	[−0.002, 0.583]	0.052
isCGM necessity	2.631	[0.026, 5.235]	0.048
isCGM satisfaction	−2.020	[−4.042, 0.002]	0.050
Constant	−24.965	[−60.118, 10.189]	0.161

Dependent variable: average scans/day with FreeStyle Libre; Independent variables: Sex, BMI, Age, Age at diagnosis, Time in target, Time above target, Illness consequences, Identity, FL necessity, FL satisfaction; Statistic methods: BACKWARDS; R^2^ = 0.457

*Β* beta, *BMI* body mass index, *isCGM* intermittently scanned continuous glucose monitoring

Men had worst isCGM adherence compared to women, with almost four fewer scans/day (*β* = −3.996; *p* = 0.011). Patients with higher BMI (*β* = −0.549, *p* = 0.017) showed to be less adherent to isCGM monitoring. On the contrary, older patients (*β* = 0.191, *p* = 0.006) and patients with a higher percentage of time in glucose target (*β* = 0.530, *p* = 0.002) demonstrated a higher isCGM monitoring adherence. Perceived isCGM necessity also positively related to monitoring frequency, showing that patients who reported higher isCGM necessity had better adherence rates (*β* = 2.631, *p* = 0.048).

## Discussion

To our knowledge, this is the first study aiming to create a model able to predict the adherence behavior to self-monitoring with intermittently scanned continuous glucose monitoring and therefore leading physicians to implement adherence strategies in patients with lower adherence patterns.

The patients enrolled in this study showed a lower glucose monitoring rate median (7 daily scans), when compared to more extensive studies like the Real-world Flash Glucose Monitoring study [13], with a median of 14 daily scans, or Flash Glucose Monitoring in Israel [19], with a median of 12 daily scans. Nevertheless, this study reaffirms the significative relation between glucose markers and monitoring rate, where patients with better HbA1C, time in target, time above target, glucose variability and average glucose had a higher glucose monitoring rate [13, 19–21].

BMI showed an important relation with isCGM adherence. Underweight/ normal BMI patients may be more concerned with following a healthier diet and practicing exercise- leading to better diabetes self-management [22] and, as acknowledged in our results, higher diabetes comprehensibility. This may explain why this population is more adherent in glucose management when compared with patients with obesity.

Previous works showed lower therapy adherence in patients with a more extended history of diabetes [3] and, consequently, the worst glucose monitoring adherence. However, in our study, there wasn’t a significant relation between monitoring frequency and TD1 duration. Nonetheless, older patients had better monitoring rates, an association also shown for the SMBG monitoring frequency [9].

Illness perceptions are shaped by past experiences and illness-related episodes [23], so it’s reasonable that patients with past year ED or hospital admissions revealed higher expectations of diabetes effects, higher perception of diabetes symptoms (data not shown), and also to be more threatened by this disease. Although this group of patients didn’t show better adherence patterns to glucose monitoring, it seems that a higher perception of illness consequences leads to better adherence.

IsCGM necessity was revealed to be an essential factor in monitoring adherence, as an extra point on the question about device necessity was associated with almost five more scans/day. Unexpectedly, and despite some studies showing positive correlations between more frequent monitoring and device satisfaction [24, 25], patients reporting less satisfaction in isCGM monitoring demonstrated better adherence. We hypothesize that fewer satisfaction levels could be related to lower precision of glucose value compared to SMBG, leading to an increase in scan rate and therefore explaining this relation.

When producing a model to predict isCGM adherence, the “patient pattern” of lower isCGM monitoring adherence is characterized as younger males with higher BMI levels, lower time in target, and less perception of isCGM necessity. The perception of isCGM necessity probably is where an intervention could be more fruitful, so it is suggested that clinicians must emphasize and create methods to enhance the need for isCGM monitoring in diabetes control.

The strengths of the present study are its real-life setting, focusing only on patients with type 1 diabetes, the knowledge of our sample sociodemographic characteristics, and the unrestricted exclusion criteria applied. All participants had the same information and support about FL management since *Centro Hospitalar de São João* provides a nurse appointment to explain and applicate the first sensor to every FL user. Clinical data was as complete and accurate as possible, considering that medical records were retrospectively retrieved when needed, and despite FL giving an estimated HbA1c value, we used laboratory values for Hba1C results to obtain the most precise measurement.

Nevertheless, this study has caveats that should be acknowledged. First, the small size of the sample. Although *Centro Hospitalar de São Joã*o is the largest hospital center in the north region of Portugal, only 158 patients are followed in the “Insulin Infusion Pump clinic”. Also, this study relies on self-report questionnaires that could jeopardize data accuracy. We tried to mitigate this factor with the questionnaires being delivered on hand by an investigator available to clarify any possible doubts of the patients.

To conclude, this study reflects the relevance of glucose monitoring adherence in disease control. Our results could help predict which patients would need more guidance from health professionals to achieve better isCGM adherence. Males of younger ages, presenting with higher BMI levels, lower time in target, and reporting lower isCGM necessity, should be closely followed and require an application of personalized adherence strategies. However, these findings must be interpreted carefully as further studies are needed to confirm the adherence patterns identified.
